# Quantitative Proteomics Reveals Protein–Protein Interactions with Fibroblast Growth Factor 12 as a Component of the Voltage-Gated Sodium Channel 1.2 (Nav1.2) Macromolecular Complex in Mammalian Brain* [Fn FN2]

**DOI:** 10.1074/mcp.M114.040055

**Published:** 2015-02-27

**Authors:** Norelle C. Wildburger, Syed R. Ali, Wei-Chun J. Hsu, Alexander S. Shavkunov, Miroslav N. Nenov, Cheryl F. Lichti, Richard D. LeDuc, Ekaterina Mostovenko, Neli I. Panova-Elektronova, Mark R. Emmett, Carol L. Nilsson, Fernanda Laezza

**Affiliations:** From the ‡Department of Pharmacology and Toxicology, University of Texas Medical Branch, 301 University Blvd, Galveston, Texas, 77555-0617;; §Neuroscience Graduate Program, Graduate School of Biomedical Sciences, University of Texas Medical Branch, 301 University Blvd., Galveston, Texas, 77555-0617;; ¶UTMB Cancer Center, University of Texas Medical Branch, 301 University Blvd., Galveston, Texas, 77555-1074;; ‖Department of Biochemistry and Molecular Biology, University of Texas Medical Branch, 301 University Blvd., Galveston, Texas, 77555-0617;; **National Center for Genome Analysis Support, Indiana University, 107 S Indiana Ave., Bloomington, Indiana, 47408

## Abstract

Voltage-gated sodium channels (Nav1.1–Nav1.9) are responsible for the initiation and propagation of action potentials in neurons, controlling firing patterns, synaptic transmission and plasticity of the brain circuit. Yet, it is the protein–protein interactions of the macromolecular complex that exert diverse modulatory actions on the channel, dictating its ultimate functional outcome. Despite the fundamental role of Nav channels in the brain, information on its proteome is still lacking. Here we used affinity purification from crude membrane extracts of whole brain followed by quantitative high-resolution mass spectrometry to resolve the identity of Nav1.2 protein interactors. Of the identified putative protein interactors, fibroblast growth factor 12 (FGF12), a member of the nonsecreted intracellular FGF family, exhibited 30-fold enrichment in Nav1.2 purifications compared with other identified proteins. Using confocal microscopy, we visualized native FGF12 in the brain tissue and confirmed that FGF12 forms a complex with Nav1.2 channels at the axonal initial segment, the subcellular specialized domain of neurons required for action potential initiation. Co-immunoprecipitation studies in a heterologous expression system validate Nav1.2 and FGF12 as interactors, whereas patch-clamp electrophysiology reveals that FGF12 acts synergistically with CaMKII, a known kinase regulator of Nav channels, to modulate Nav1.2-encoded currents. In the presence of CaMKII inhibitors we found that FGF12 produces a bidirectional shift in the voltage-dependence of activation (more depolarized) and the steady-state inactivation (more hyperpolarized) of Nav1.2, increasing the channel availability. Although providing the first characterization of the Nav1.2 CNS proteome, we identify FGF12 as a new functionally relevant interactor. Our studies will provide invaluable information to parse out the molecular determinant underlying neuronal excitability and plasticity, and extending the relevance of iFGFs signaling in the normal and diseased brain.

Voltage-gated sodium channels (Nav)^1^ are transmembrane proteins consisting of a pore-forming α subunit (Nav1.1-Nav1.9) and one or more accessory β-subunits (β_1_–β_4_) (1–3). Predominately clustered at the axonal initial segment (AIS), the α subunit alone is necessary and sufficient for channel assembly and the initiation and propagation of action potentials following membrane depolarization (4). Although the α subunit is functional on its own, it is the transient and stable protein–protein interactions that modulate subcellular trafficking, compartmentalization, functional expression, and fine-tune the channel biophysical properties (5–9). Thus, the Nav channel and the protein constituents that comprise the protein–protein interaction network are all part of a macromolecular complex that modulates the spatiotemporal dynamics of neuronal input and output playing a critical role in synaptic transmission, signal integration, and neuronal plasticity. Perturbations in this protein–protein interaction network can lead to deficits in neuronal excitability, and eventually neurodegeneration and cell death (10–15).

Given the relevance of these interactions for the native channel activity and its overall role in controlling brain circuits, it is increasingly important to uncover these associations. Antibody-based affinity purification (AP) combined with mass spectrometry (MS) is widely used for the enrichment and analysis of target proteins and constituents of their protein–protein interactions as it can be performed at near physiological conditions and preserves post-translational modifications relevant to protein complex organization (16–19). Differential mass spectrometry provides an unbiased method for the efficient, MS-based measurement of relative protein fold changes across multiple complex biological samples. This technology has been successfully applied to a number of ion channels (20–26), but—to the best of our knowledge—not to the study of any member of the Nav channel family. Using a target-directed AP approach combined with quantitative MS, we identified proteins constituting the putative interactome of Nav1.2, one of three dominant Nav channel isoforms in the mammalian brain, from native tissue (1, 2, 4, 8). Among these putative interactors, the fibroblast growth factor 12 (FGF12), a member of the intracellular FGF family (5, 13, 14), stood out as one of the most abundant coprecipitating proteins with ∼30-fold enrichment over other interactors. With a combination of confocal microscopy in brain tissue, reconstitution of the interactor in a heterologous systems and electrophysiological assays, we provide validation for FGF12 as a *bona fide* relevant component of the Nav1.2 proteome and a modulator of Nav1.2-encoded currents. Altogether, the identified channel/protein interaction between FGF12 and Nav1.2 provides new insights for structural and functional interpretation of neuronal excitability, synaptic transmission, and plasticity in the normal and diseased brain.

## MATERIALS AND METHODS

### 

#### 

##### Chemicals and Reagents

LC-MS grade acetonitrile and water were from J.T. Baker (Philipsburg, NJ). Formic acid, tris (2-carboxyethyl) phosphine (TCEP), and Protein-A/G MagnaBind® beads were from Pierce (Rockford, IL). Iodoacetamide (IAA), BSA, aprotinin, and EDTA were obtained from Sigma-Aldrich (St. Louis, MO). Sodium chloride (NaCl) and sodium fluoride (NaF) were supplied by BDH (West Chester, PA). Protease inhibitors antipain, leupeptin, benzamidine, pepstatin, and sodium azide (NaN_3_) as well as Triton X-100 were purchased from Amresco (Solon, OH) and PMSF from CalBiochem (Darmstadt, Germany). Sequencing grade Lys-C and trypsin were from Roche (Mannheim, Germany) and Promega (Madison, WI), respectively.

##### Animals

Adult Sprague-Dawley rats were purchased from Harlan Laboratories (Indianapolis, IN). Rats were sacrificed via isoflurane exposure followed by decapitation. Dissected whole brains were immediately frozen in liquid nitrogen vapor and stored in −80 °C until use.

##### Crude Membrane Extract

Adult rat brains were homogenized as previously described (27) in 0.3 m sucrose/10 mm sodium phosphate monobasic with EDTA (pH 7.4) at a final concentration of 1 mm containing the following protease inhibitors: leupeptin (1 μg/ml), aprotinin (1 μg/ml), pepstatin (1 μg/ml), and PMSF (1 mm). The homogenate was centrifuged for 10 min at 3000 × *g* at 4 °C to remove cellular debris. The supernatant was centrifuged for 90 min at 45,000 × *g* at 4 °C and the pellet (crude membrane extract; CME) was resolubilized in homogenization buffer. Protein concentrations were measured by BCA (Pierce, Rockford, IL).

##### Affinity Purification of Nav1.2

CME was diluted 1:10 (∼1 mg/ml) in buffer containing: 1% Triton X-100, 0.15 m NaCl, 1 mm EDTA, 10 mm sodium azide, 10 mm Tris-HCl (pH 8.0), 2 mm NaF, 1 mg/ml BSA, 1.5 μg/ml aprotinin, 10 μg/ml antipain, 10 μg/ml leupeptin, 0.1 mg/ml benzamidine, and 1 mm PMSF on a tube rotator for 30 min at 4 °C (28). Next the samples were centrifuged at 16,000 × *g* for 30 min at 4 °C to remove the insoluble fraction. Samples were incubated overnight at 4 °C with 15 μg of immobilized mouse monoclonal antibody Nav1.2 (K69/3; UC Davis/NIH NeuroMab Facility, CA) or control mouse IgG (sc-2025; Santa Cruz Biotechnology, Santa Cruz, CA). Beads were washed three times in (1 ml each) the same buffer without BSA and eluted with 0.2 m glycine at 25 °C for 15 min. Protein concentrations were measured with Nanodrop (ThermoFisher, Wilmington, DE). For transfected HEK-293 cells stably expressing rat Nav1.2 α subunit and *myc-Fgf12b*, or *myc-sprouty*, cells were treated and lysed as previously described (5) for co-immunoprecipitation experiments. The *myc-Fgf12* (*myc-fgf12–1b*) and *myc-sprouty* fusion constructs were a gift from Dr. David Ornitz (Washington University in St. Louis, MO).

##### Gel Electrophoreses and Western blot Analyses

Eluents were titrated with neutralization buffer (1 m Tris, pH 9.5; 1/4 of the elution volume) to physiological pH and boiled in 2× loading buffer for 5 min and separated on 4–20% polyacrylamide gels (BioRad, Hercules, CA). Proteins were transferred to a nitrocellulose membrane (Millipore, Bedford, MA) for 2 h at 75 V and blocked with 5% nonfat dry milk in Tris-buffered saline with 0.1% Tween-20 (TBS-T) for 1 h at room temperature. Membranes were probed with mouse antiPanNav channel (1:1000; Sigma Aldrich, St. Louis, MO), and anti-c-Myc (1:1000; Santa Cruz Biotechnology. Santa Cruz, CA) in blocking buffer overnight at 4 **°**C. Blots were washed with TBS-T (two times for 15 min), and probed with horse anti-mouse secondary antibody (1:10,000) conjugated to horseradish peroxidase (Vector Lab, Burlingame, CA) and detected with ECL Advance Western blotting Detection kit (GE Healthcare, Piscataway, NJ). Proteins were visualized using FluorChem® HD2 System with AlphaView 3.1 software (ProteinSimple, Santa Clara, CA).

##### Reduction, Alkylation, and Digestion

Eluted proteins were titrated to physiological pH and precipitated with the 2D Clean-Up Kit (GE Healthcare, Piscataway, NJ). The precipitated protein was resuspended in 8 m urea, 25 mm ammonium bicarbonate (pH 8), reduced with 5 mm TCEP (pH 8.0) for 30 min at room temperature, and alkylated with 10 mm IAA for 30 min at room temperature in the dark. Proteins were digested overnight with Lys-C 1:100 (w/w) at 37 **°**C, and subsequently with trypsin 1:50 (w/w) overnight at 37 **°**C. Samples were dried to completeness in a SpeedVac and stored at −80 **°**C until analysis.

##### Mass Spectrometry

Samples were resuspended in 0.1% FA/5% ACN (v/v). Each sample was analyzed in a block-randomized fashion (29) by nanoLC-MS/MS on a hybrid mass spectrometer consisting of a linear quadrupole ion trap and an Orbitrap (LTQ-Orbitrap Elite, Thermo Fisher Scientific) in positive ion mode. Separations were performed using an online EasyLC-1000 nanoflow HPLC (Proxeon Biosystems, Odense, Denmark). Peptides were loaded on to a 100 μm ID × 2 cm C_18_ trap column (ThermoFisher). The chromatographic separation was performed on PicoFrit® (360 μm OD × 75 μm ID ×15 μm) column packed with 10 cm ProteoPep II (5 μm, 300 Å, C_18_, New Objective, Woburn, MA) at 250 nL/min. Mobile phases were 0.1% FA in water (A) and 0.1% FA in ACN (B). Samples were eluted from the column with 5% solvent B for 5 min. After 5 min the gradient was ramped to 35% B over 140 min and further increased to 95% B over 20 min and held for an additional 15 min. Total run time, including column equilibration, sample loading, and analysis was 202 min.

The mass spectrometer was operated in data-dependent mode to automatically switch between MS and MS/MS acquisition. The survey scans (*m*/*z* 350–2000) (MS) were acquired in the Orbitrap at high resolution (120,000 at *m*/*z* 400) in profile mode, and the MS/MS spectra were acquired in the linear ion trap at low resolution, in centroid mode using XCalibur, version 2.0.7 (Thermo Fisher Scientific). Ion injection times for the MS and MS/MS scans were 500 ms and 150 ms, respectively. The automatic gain control targets were set to 1 × 10^6^ for MS in the Orbitrap and 1 × 10^4^ for MS/MS in the LTQ. The 15 most abundant precursor ions above a 10,000 counts threshold from each MS scan were sequentially isolated and fragmented in the LTQ using CID (isolation width 2.0 Da, default charge state of four, normalized collision energy 35%, activation *Q* 0.250, and activation time 30 ms). Dynamic exclusion (±10 ppm relative to precursor ion *m/z*) was enabled with a repeat count of one, maximal exclusion list size of 500, and an exclusion duration of 60 s. Monoisotopic precursor selection (MIPS) was enabled and unassigned and singly charged ions were rejected. The general mass spectrometric conditions were as follows: spray voltage 2.2 kV, 40% S-lens, and capillary temperature 275**°**C. Spectra were acquired using XCalibur, version 2.0.7 (ThermoFisher).

##### Data Processing

MS files (.raw) were imported into Progenesis LC-MS (version 4.1; Nonlinear Dynamics, Newcastle upon Tyne, U.K) for peak list generation and *m*/*z* and retention time alignment using a proprietary algorithm and manual landmarks with one sample set as the reference as previously described (30). This was followed by exclusion of features with one charge or more than six charges. The top five spectra for each feature were exported as a combined .mgf file and searched with MASCOT (version 2.1.6), X!Tandem (version 2013.06.15), and PEAKS (version 6, Bioinformatics Solutions Inc., Waterloo, ON) against a merged UniprotKB/SwissProt RatMouse database of canonical sequences (July 2013; 24,541 entries) appended with the cRAP contaminant database (February 2012 version, The Global Proteome Machine, www.thegpm.org/cRAP/index.html). Precursor ion mass tolerance was set to 10 ppm and fragment mass tolerance was 0.8 Da. A maximum of two missed cleavages were allowed using trypsin as the endoprotease; carbamidomethylation of cysteine and oxidation of methionine were set as fixed and variable modifications, respectively. Mascot, X!Tandem, and PEAKS searches were combined (using PEAKS inChorus), with a 1% false discovery rate cutoff for all search engines. Protein identifications were annotated at ≥ 95% probability and imported into Progenesis LC-MS for conflict resolution, which was performed manually to ensure that a single peptide sequence was assigned to each feature by removing lower scoring peptides. Proteins with MS spectra significantly differentially expressed between conditions (Nav1.2 *versus* IgG) at 80% power, 10,000 minimum intensity threshold, and a fold change ≥ 2 with no MS/MS spectra were exported as an inclusion list with *m/z* and retention time windows. Samples were re-run in a block-randomized fashion as described above with the following exceptions: FT preview scan and dynamic exclusion were turned off, and precursor ion threshold was set to 1000 counts. Spectra were imported into Progenesis and aligned with original data sets, searched, and annotations combined with previous data. Proteins identified as exogenous contaminations such keratin or immunoglobulin were eliminated. The mass spectrometric data have been deposited in ProteomeXchange (http://proteomecentral.proteomexchange.org) via the PRIDE partner repository (31) with the data set identifier PXD000719.

##### Data Analysis

Annotations from combined database searching at ≥ 95% probability were exported with raw abundances to Excel and processed for differential (label-free) quantification. First, all peptides containing modification other than oxidation of methionine or carbamidomethylation of cysteine were removed. Second, all proteins not identified by at least two unique peptides were removed. The table of peptide intensities per LC run was imported into a custom SAS script for analysis (supplemental Table S1). The intensity of each peptide ion species was standardized across all measures of that peptide species. Of the 64,262 possible intensity values (23 runs × 2794 peptide ion species), only 1363 or 2.121% were zero values. These values were assumed to be missing at random and were excluded from the analysis. All calculations were done using SAS PROC MIXED with restricted maximum likelihood estimations (SAS Institute, Cary, NC), and type 3 sums of squares (where appropriate). A hierarchical linear model was used to test for differences in mean intensity between the Nav and control samples, while allowing each biological replicate to have its own overall mean, and allowing each technical replicate within the biological replicates to have its own mean. Each *p*-value of the resulting 370 F tests was corrected for multiple testing with a FDR of 0.05 (32). Next, the same model was run on the log_2_-converted raw intensities. The difference in estimated mean between Nav and control in these tests was taken as an estimate of the overall fold change within the treatment. Significance values adjusted for multiple hypothesis testing (*q*-value) for each protein confidently identified combined with fold change to improve separations between false positives and genuine interacting partners (32). Relative sequence coverage (SC) for Nav1.2 (P04775) was calculated as previously described (22). The number of identified amino acids passing our filters (*N_i_*) was divided by the sum of identified amino acids and MS-accessible (6–25 residues) but not identified amino acids (*N_an_*) in the respective UniProtKB database; that is, *SC* = (*N_i_*)/(*N_i_* + *N_an_*). Calculations for *N_an_* were made for MS-accessible peptides generally and for MS-accessible peptides excluding those belonging to predicted transmembrane domains. All identified proteins with their respective numbers of unique peptides and sequence coverage (%) before quantification are listed in supplemental Table S2. All identified proteins with their respective cumulated abundances from each experimental condition (*i.e.* Nav and control), *p*-value, *q*-value, and log_2_ fold change are listed in supplemental Table S3.

##### Immunohistochemistry

Was performed as previously described (5). Briefly sagittal sections were serially cut and mounted on Superfrost® glass microscope slides (Fisher Scientific, Waltham, MA) and left to dry overnight at room temperature. Samples were washed, fixed in −20 °C acetone, and incubated overnight with the following primary antibodies: mouse antiFGF12 (monoclonal 1:100; UC Davis/NIH NeuroMab Facility, CA), rabbit antiPanNav (polyclonal 1:100; Sigma, St. Louis, MO), mouse antiNav1.2 (monoclonal 1:100; UC Davis/NIH NeuroMab Facility, CA), and chicken anti-β-IV-spectrin (polyclonal 1:5000, gift from Dr. M. Komada, Tokyo Institute of Technology, Tokyo, Japan). Samples were then washed and incubated for 1 h with Alexa 488-conjugated goat-anti-mouse and Alexa 568-conjugated goat-anti-rabbit (1:200, Molecular Probes, Eugene, OR) for FGF12 and PanNav antibodies. Mouse FGF12, Nav1.2, and β-IV-spectrin antibodies were probed with isotype specific Alexa 488-conjugated goat-anti-mouse IgG_1_, 568-conjugated goat-anti-mouse IgG_2a_, and Alexa 647-conjugated goat-anti-chicken, respectively (1:200). Samples were then mounted on glass slides with Prolong Gold antifade reagent (Invitrogen, Carlsbad, CA). Confocal images were acquired with a Zeiss LSM-510 Meta confocal microscope with a Plan-Apochromat 20× air objective (0.75 NA), 40× (0.95-watt corr) water objective, a C-Apochromat 63× (1.2-watt corr) water objective, and a Plan-Apochromat 63× (1.4 NA) oil-immersion objective. Multitrack acquisition was performed with excitation lines at 488 nm, 543 nm, and 633 nm for Alexa 488, Alexa 568, and Alexa 647 respectively. Respective emission filters were band-pass 505–530 nm, 560–615 nm, and low pass 650 nm. The optical slices were 4–1 μm and 0.8 μm; Z-stacks were collected at *z*-steps of 2–0.5 μm and 0.4 μm with a frame size of 512 × 512 and 1024 × 1024, pixel time of 2.5 μs, pixel size 1.2 × 1.2 μm (20x), 0.16 × 0.16 μm (40x), or 0.39 × 0.39 μm and 0.1 × 0.1 μm (63x) and an 4–8 frame Kallman-averaging.

##### Cell Culture and Transient Transfections

HEK-293 cells stably expressing rat Nav1.2 (HEK-Nav1.2 cells) were maintained as previously described (5). Nav1.2-expressing HEK cells were transiently transfected with either *Fgf12b-GFP* or *Gfp* at 90–100% confluency using Lipofectamine 2000 (Invitrogen, Carlsbad, CA), according to manufacturer's instructions. Cells were then dissociated and replated at low-density, prior to the patch-clamp recording.

##### Electrophysiology and Data Analysis

Recordings were performed at room temperature (20–22 °C) 12–18 h post-transfection using a MultiClamp 700B amplifier (Molecular Devices, Sunnyvale, CA). The recording solutions were as follows: extracellular (mm): 140 NaCl, 3 KCl, 1 MgCl_2_, 1 CaCl_2_, 10 HEPES, 10 glucose, pH 7.3; intracellular: 130 CH_3_O_3_SCs, 1 EGTA, 10 NaCl, 10 HEPES, pH 7.3. Membrane capacitance and series resistance were estimated by the dial settings on the MultiClamp 700B amplifier (Molecular Devices). Capacitive transients and series resistances were compensated electronically by 70–80%. Data were acquired at 20 kHz and filtered at 2.2 kHz prior to digitization and storage. All experimental parameters were controlled by pCLAMP 9 software (Molecular Devices) and interfaced to the electrophysiological equipment using a Digidata 1322 analog-digital interface (Molecular Devices). Voltage-dependent inward currents were evoked by depolarizations to test potentials between −60 mV and +50 mV from a holding potential of −90 mV. Steady-state (fast) inactivation of Nav channels was measured with a paired-pulse protocol. From the holding potential, cells were stepped to varying test potentials between −110 mV and 20 mV (prepulse) prior to a test pulse to −10 mV. Current densities were obtained by dividing Na^+^ current (I_Na_) amplitude by membrane capacitance. To inspect the quality of the recordings, current-voltage relationships were generated by plotting current density as a function of the holding potential. Conductance (*G_Na_*) as calculated by the following equation:

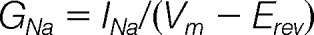
 where *I_Na_* is the current amplitude at voltage *V_m_*, and *E_rev_* is the Na^+^ reversal potential.

Steady-state activation curves were derived by plotting normalized *G_Na_* as a function of test potential and fitted using the Boltzmann equation:

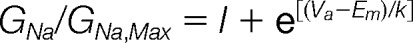
 where *G_Na,Max_* is the maximum conductance, *V_a_* is the membrane potential of half-maximal activation, *E_m_* is the membrane voltage and k is the slope factor. For steady-state inactivation, normalized current amplitude (*I_Na_/I_Na,Max_*) at the test potential was plotted as a function of prepulse potential (*V_m_*) and fitted using the Boltzmann equation:

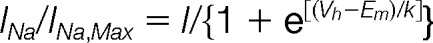
 where *V_h_* is the potential of half-maximal inactivation and k is the slope factor. Data analysis was performed using Clampfit 9 software (Molecular Devices) and Origin 8.6 software (OriginLab, Northampton, MA). Results were expressed as mean ± S.E. The statistical significance of observed differences among groups was determined by either one-way ANOVA or Kruskal-Wallis. A *p* ≤ 0.05 was regarded as statistically significant. Bonferroni or Dunn's tests were used for the *post hoc* analysis.

## RESULTS

### 

#### 

##### Affinity Purification and MS Analysis of Nav1.2 Co-Purified Proteins

**Fig. 1.**
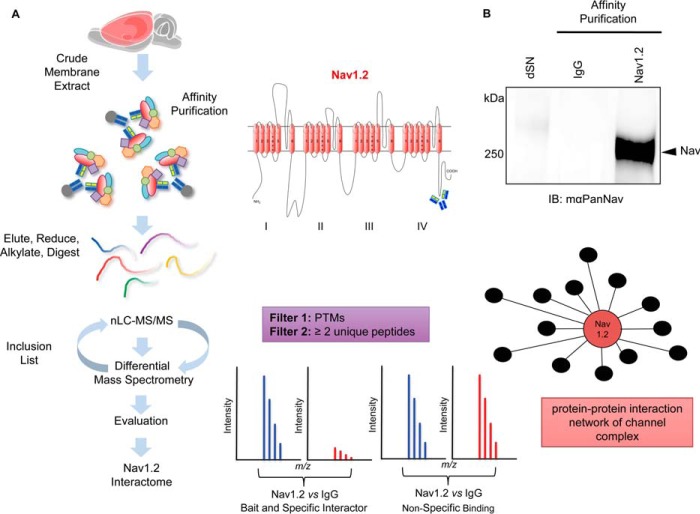
**Proteomic workflow for downstream nLC-MS/MS and differential MS quantification.** *A*, Workflow outlining experimental procedures and nLC-MS/MS data acquisition for analysis for identification of Nav1.2 protein–protein interactions as detailed in the text. (*center inset*). The predicted tertiary structure of Nav1.2 is shown with antibody and epitope site. *B*, Representative Western blot of affinity purified Nav1.2 channel complex from adult rat brain. Nav1.2 and control probed with mouse antiPanNav antibody show the Nav1.2 channel is present in the Nav1.2 pulldown, but absent in the control. The depleted supernatant (dSN) reveals > 80% depletion of Nav1.2 protein was achieved.

The proteomic workflow used to characterize Nav1.2 and its protein constituents is outlined in Fig. 1*A*. Crude membrane extracts were prepared from whole brain of adult rats as previously described (28). Monoclonal antiNav1.2 antibody K69/3 targeted against the C-terminal domain (1885–2005) of Nav1.2, which has been independently tested and validated in previous proteomic studies (28), was used for affinity purification of the channel complex. We performed this study with anti-Nav1.2 antibody to build confidence in the isoform specificity of the interactors in light of the lack of a viable knockout animal model for Nav1.2, which would serve as a guard against antibody off-target effects (22, 24). To filter for false positives a nonspecific mouse immunoglobulin G (mIgG) was used for control samples. Western blot analysis of the affinity-purified proteins revealed robust and specific Nav1.2 enrichment from rat brain as evidenced by the absence of signal in the control (Fig. 1*B*).

To identify the native Nav1.2 protein interactors, peptides from total AP eluents were analyzed using nanoLC-MS/MS. High-resolution MS spectra provided *m*/*z*, retention time, charge state, and amount of precursor ions from four technical analyses of Nav1.2 and control per biological replicate. Mass spectra of precursor ions were aligned based on *m*/*z* and retention times and quantified based on total isotopic peak volume (sum of *m*/*z* signal intensities for a given peptide over retention time) (22, 26). MS peptide features were annotated by database searches of tandem mass spectrometry (MS/MS) spectra and subjected to a two-tiered filter as described in “Materials and Methods.” The peptides confidently identified in our analysis were plotted based on mass error (ppm) resulting in a Gaussian curve distribution (supplemental Fig. S1*A*). All peptides annotated at ≥ 95% probability fell within a mass error range of ±5 ppm, with the overwhelming majority falling within a mass error range of ±3 ppm. Principal component analysis of all biological and technical replicates in both Nav and control APs demonstrate clear separation based on experimental condition (PCA1) and biological replicates (PCA2 and PCA3) with tight clustering of technical replicates (supplemental Fig. S1*B*).

**Fig. 2.**
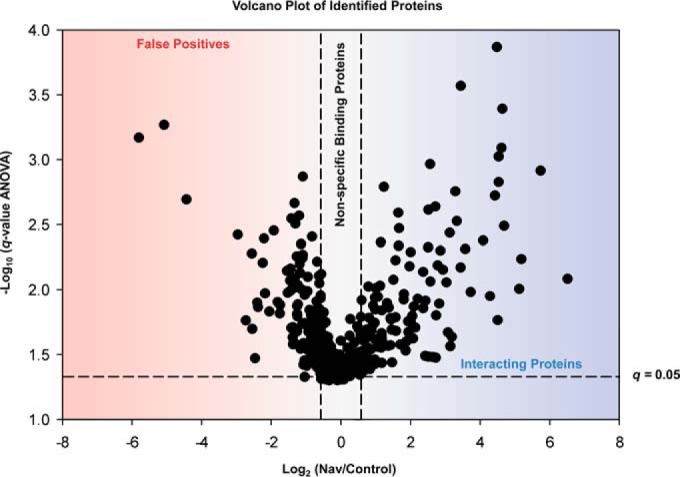
**Determination of putative interaction partners.** Logarithmic ratios of Nav *versus* control experiments performed in quadruplicate are plotted against the negative logarithmic *q*-value (where *q* ≤ 0.5 is considered significant; horizontal line) of the hierarchical linear model with random biological replicates nested within the two antibody treatment levels. Nonspecific binding proteins have a ratio around 1:1 and are located in the center; vertical lines designate the logarithmic ratio of a ±1.5-fold change. Proteins located in the blue area are considered putative interaction partners whereas proteins in the red area are false positives as no proteins are expected be more abundant in the control. All proteins are listed in supplemental Table S4 with their respective *q*-values and log_2_ fold change.

Utilizing the workflow outlined in Fig. 1*A*, we obtained >70% protein sequence coverage of Nav1.2 (supplemental Fig. S2) (Materials and Methods) and 75% when excluding MS-accessible peptides (*i.e.* 6–25 amino acids) belonging to predicted Nav1.2 transmembrane domain alpha helices, and identified 107 coprecipitating proteins as putative constituents of the Nav1.2 interactome in rat brain. We evaluated our data by plotting the logarithmic ratios of Nav *versus* control affinity-purified proteins against the negative log_10_ *q*-value (Fig. 2). We applied significance (dashed horizontal lines) and fold change thresholds (dashed vertical lines), to help in distinguishing between proteins that comprise putative constituents of the Nav1.2 channel complex, proteins that bound nonspecifically to the matrix, and potential false positives (34–36).

Of the putative interactors we identified (supplemental Table S4), some have been found to precipitate with other Nav channel isoforms (*e.g.* 14–3-3η with Nav1.5) (37), whereas many others have been implicated previously in the Nav1.2 interactome, such as FGF14 (5, 13, 14) and β subunits (β1–4), crucial Nav interactors implicated in diseases (3, 11). In our data set, we identified the noncovalently bound β1 subunit, but we confidently identified only one unique β1 subunit peptide and therefore excluded it from further analysis. On the other hand, the β2 subunit (*q* = 0.0012), which is covalently linked to Nav through disulfide bonds, passed our threshold criteria to be declared Nav1.2 interactor (supplemental Table S4). Other relevant proteins in our data set included Ankyrin-3 (or Ankyrin-G; *q* = 0.0214) a structural scaffolding protein interacting with Nav channels at the AIS (38, 39) (supplemental Table S4), calmodulin (*q* = 0.0019), and multiple isoforms of CaMKII (supplemental Table S4). The Nav1.2 channel isoforms contain high affinity calmodulin (CaM)-binding IQ domain at the C terminus (residues 1901–1927 for Nav1.2), which has been implicated in regulation of the channel biophysical properties (40–42). These results, which recapitulate previous independent studies, add validation to our AP-MS data set.

**Fig. 3.**
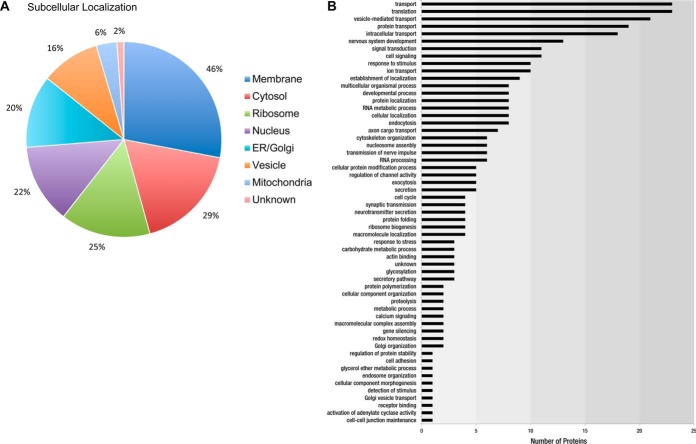
**Analysis of Nav1.2 coprecipitating proteins in mammalian brain.** *A*, Subcellular localization of identified Nav1.2 interacting proteins *B*, Nav1.2 protein constituents categorized according to biological function using Gene Ontology and UniProtKB.

Subcellular localization analysis of interactors using public databases (UniprotKB/SwissProt, EMBL-EBI, GO, and PubMed) showed nearly half of all identified proteins reside at the plasma membrane (46%) (Fig. 3*A*). Other subcellular compartments included cytosol (29%), ribosome (25%), nucleus (22%), ER/Golgi (20%), intracellular and synaptic vesicles (16%) and mitochondria (6%), with only 2% of all proteins lacking annotation of subcellular localization (supplemental Table S5). Analysis of biological function of identified protein constituents is shown in Fig. 3*B*.

##### Nav1.2 and Intracellular FGFs

Intracellular FGFs (FGF11–14 or FHF1–4) are integral components of the Nav macromolecular complex in the CNS that modulate channel currents (5, 13, 14, 43). Our previous studies found FGF14 strongly colocalized with Nav channels predominantly in the dentate gyrus and CA3 region of the hippocampus in native tissue. This interaction is phosphorylation-dependent and is controlled by a network of kinases converging on glycogen-synthase kinase 3 (GSK-3), such that inhibition of GSK3 results in subcellular redistribution of the native FGF14:Nav complex and reduction in intrinsic excitability (44). In this study, we found both FGF14 and FGF12 in Nav1.2 purifications. However, only one unique peptide corresponding to FGF14 was identified in our AP-MS data set and was not used for quantification. FGF14's more specific regulatory effect for other Nav isoforms such as Nav1.6 and Nav1.1 might have resulted in low overall abundance in the Nav1.2 complex (supplemental Fig. S3*A*–3*B*). On the other hand, FGF12 met all our criteria and was over 30-fold enriched in Nav1.2 purifications (*q* = 0.0099; supplemental Table S4) relative to control purifications with mouse IgG.

##### FGF12 and Nav Channels Form a Complex in Native Tissue

**Fig. 4.**
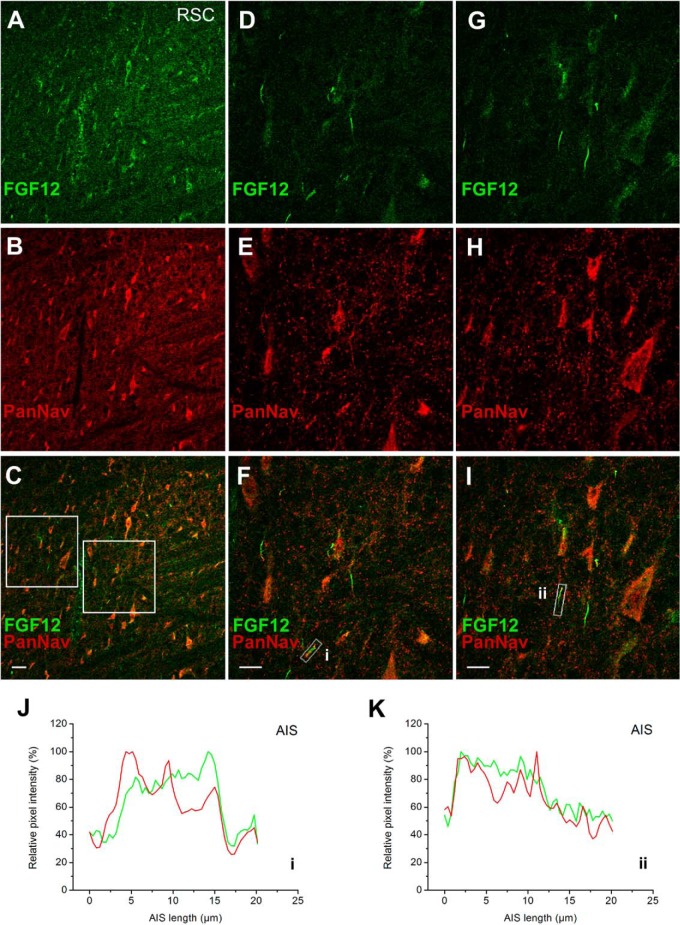
**Distribution of the subcellular colocalization of FGF12 and Nav channel in mammalian brain.** *A, D*,and *G*, Confocal images of principal neurons in the retrosplenial cortex (RSC) stained with a mouse monoclonal antiFGF12 antibody, visualized with Alexa 488 secondary antibody. *B, E*,and *H*, antiPanNav α subunit antibody visualized with Alexa 568 secondary antibody. *C, F*,and *I*, Overlaid images of the green (FGF12) and red (PanNav) are shown. Boxed area to the *left* in panel *C*, highlights region used for higher magnification in panels *D–F*. Boxed area to the *right* in panel *C* highlights region used for higher magnification in panels *G–I*. The boxed regions in *F* and *I* highlight two representative AIS (i,ii) with pixel intensity profiles of both channels shown below *J* and *K. A–C*, represent images taken at 20× objective (air) and *D–I* represent images taken at 63× objective. *Scale bar*: 50 μm in *A–C* and 20 μm in *D–I*.

We previously demonstrated the subcellular distribution of FGF14 expression in mammalian brain (5). Previous studies have shown the relative distribution of FGF12 protein and RNA in the brain (45, 46). However, information on the subcellular distribution of native FGF12 in neurons of the CNS is lacking. Therefore, we used confocal microscopy as an orthogonal method to demonstrate colocalization of FGF12 with Nav channels in native tissue. In initial studies, we fixed and probed rat brain slices with a rabbit antibody against PanNav channels and mouse monoclonal antibody against FGF12 (IgG_1_). Confocal analysis revealed a strong colocalization of FGF12 and Nav channel at the AIS (Fig. 4*A*–4*K*) with less intense, but distinct overlapping distribution of the two proteins in the somato-dendritic compartment of neurons in the retrosplenial cortex (Fig. 4*A*).

**Fig. 5.**
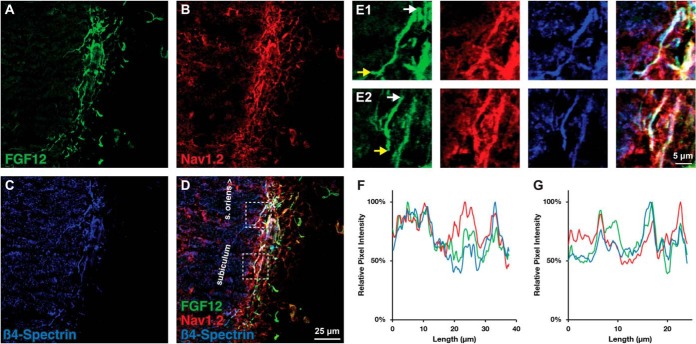
**Distribution of the subcellular colocalization of FGF12 and Nav1.2 channel in mammalian brain.** *A*, Confocal images of principal neurons of the subiculum stained with antiFGF12 antibody, visualized with Alexa 488 secondary antibody. *B*, AntiNav1.2 α subunit antibody visualized with Alexa 568 secondary antibody. *C*, anti-β-IV-spectrin antibody visualized with Alexa 647 secondary antibody. *D*, Overlaid images of the green (FGF12), red (Nav1.2), and blue (β-IV-spectrin) are shown. Boxed areas in panel *D* highlights region used for higher magnification in panels *E*1 and *E*2. Boxed (*upper*) area in panel *D* highlights region used for higher magnification in panel *E*1. Boxed (*lower*) area in panel *D* highlights region used for higher magnification in panel *E2. E*1 and *E*2) Left to right, panels demonstrating FGF12, Nav1.2, β-IV-spectrin, and merge channels in two representative AIS. White arrow is where tracing began and yellow arrow is where tracing ended. Pixel intensity of both AIS is shown below *F–G. A–D* represent images taken at 40× objective (water) and (*E*1–*E*2) represent images taken at 40× objective with 3× zoom. *Scale bar*: 25 μm in (*A–D*) and 5 μm in (*E*1–*E*2).

It is notable that Nav1.2 is the most abundant isoform found at the proximal region of the AIS, whereas other neuronal channels, such as Nav1.6 have more specialized locations (47–49). Thus, the AIS staining resulting from a PanNav antibody (Fig. 4) might likely represent for the vast majority the Nav1.2 isoform immunoreactivity. However, to further validate our findings, we extended our immunohistological evaluations using a mouse monoclonal antibody against Nav1.2 (IgG_2a_) along with the FGF12 and a chicken β-IV-spectrin antibody (an AIS marker). The FGF12:Nav1.2 complex was visualized with Alexa-conjugated isotype-specific secondary antibodies (IgG_1_ and IgG_2a_, respectively) along with conventional Alexa-conjugated anti-chicken. Confocal analysis confirmed a strong colocalization of FGF12 and Nav1.2 at the AIS in the subiculum of the hippocampal formation (Fig. 5*A*–5*D*) with fluorescence intensity profiles of all three channels at selected AIS (Fig. 5*E*1–*E*2) shown below (Fig. 5*F*–5*G*). Taken together, these results identify native FGF12 as a component of the Nav1.2 channel complex, confirming our mass spectrometry results.

##### Complex Formation of FGF12 with Nav1.2 and CaMKII-dependent Modulation of the Na1.2 Current Amplitudes

**Fig. 6.**
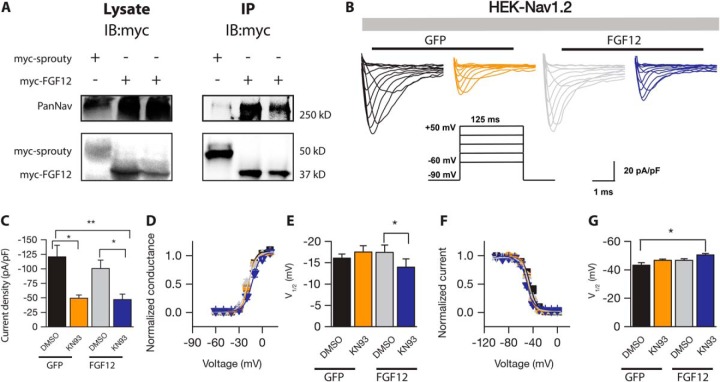
**FGF12B interaction and modulation of Nav1.2 channels.** *A*, Western blots of lysates from HEK-Nav1.2 cells cotransfected with myc-FGF12 or myc-sprouty (as negative control) were probed with PanNav and myc antibodies. The co-immunoprecipitation of Nav1.2 with myc-tagged FGF12, but not myc-sprouty in HEK-Nav1.2 cells confirms complex formation between the proteins. *B*, Representative traces of voltage-gated Na^+^ currents (*I*_Na_) recorded from HEK-Nav1.2 cells transiently expressing GFP or GFP-FGF12B in response to voltage steps from −60 mV to +50 mV from a holding potential of −90 mV (*inset*). Only selected current traces in response to voltage steps are shown. GFP-expressing cells were treated with either 0.05% DMSO (*black traces*) or with 5 μm of KN93 (orange traces), whereas GFP-FGF12B-expressing cells were treated with either 0.05% DMSO (*gray traces*) or with 5 μm KN93 (*blue traces*). *C*, Bar graphs representing peak current densities measured in individual cells HEK-Nav1.2 cells expressing either GFP (treated with 0.05% DMSO; *black bar*) or GFP (treated with 5 μm KN93; orange bar), and FGF12B-GFP (treated with 0.05% DMSO; *gray bar*), or GFP-FGF12 (treated with 5 μm KN93; *blue bar*). Data are mean ± S.E. Pharmacological inhibition of KN93 in cells expressing either GFP suppress peak current densities (at 0 mV) in comparison with DMSO-treated control (*p < 0.05, one-way ANOVA Kruskal Wallis, *post hoc* Dunn's method) or GFP-FGF12B suppress peak current densities (at 0 mV) in comparison with DMSO-treated control (*p < 0.05, one-way ANOVA Kruskal Wallis, *post hoc* Dunn's method). Change in peak current density between cells expressing GFP (treated with DMSO) and FGF12B (treated with 5 μm KN93) is shown as **p < 0.01, one-way ANOVA Kruskal Wallis, *post hoc* by Dunn's method. Voltage dependence of *I*_Na_ activation, *D*, V_1/2_ (half-maximal voltage activation, *E*, and voltage dependence of *I*_Na_ inactivation, *F*, V_1/2_ (half-maximal voltage inactivation) steady-state inactivation, *G*, were measured as described under Experimental Procedures and mean ± S.E. values are plotted as a function of the membrane potential. The activation and inactivation data were fitted with the Boltzmann function as described under “Experimental Procedures.” The fitted parameters are provided in Table I. Changes in the V_1/2_ of activation and steady-state inactivation between cells expressing GFP-FGF12B (treated with KN93) in comparison with DMSO-treated control group (for activation, Panel *D*, *p < 0.05, one-way ANOVA, *post hoc* Bonferroni), and (for steady-state inactivation, Panel *F*, *p < 0.05, one-way ANOVA, *post hoc* Bonferroni) are shown.

First, we aimed to independently reconfirm our AP-MS findings, namely Nav1.2 and FGF12 complex formation, in HEK-Nav1.2 cells by co-immunoprecipitation experiments. We found that the immunoprecipitation of myc-FGF12 with anti-myc antibodies also co-immunoprecipitated the Nav1.2 channel stably expressed in HEK-293 cells, whereas myc-sprouty immunoprecipitation with anti-myc antibody failed to recover Nav1.2 (Fig. 6*A*).

Previous studies have shown that other iFGFs regulate amplitude and voltage-dependence of Nav-encoded Na^+^ currents in cell lines (50–52) and CaMKII has a documented role in modulation of cardiac Nav1.5 channels with CaMKII activation resulting in a hyperpolarizing shift in steady state inactivation (53). Furthermore, studies from our group indicate that the FGF14:Nav channel complex assembly and the channel functional modulation by the factor are phosphorylation-dependent indicating an interplay between kinases and iFGFs (5, 44, 54). Through our AP-MS strategy, in addition to FGF12, we identified CaMKII (supplemental Table S4). Of the existing CaMKII isoforms, CaMKII-α (*q* = 0.00097), -β (*q* = 0.0170), and -γ (*q* = 0.0250) were found significant Nav1.2 channel interactors, whereas CaMKII-δ was excluded because of insufficient number of peptides. Thus, we posited that the CaMKII pathway could influence the functional modulation of Nav1.2-encoded currents.

To test this hypothesis, we designed a 2 × 2 study to evaluate the role of FGF12B in regulating amplitude and biophysical properties of Nav1.2-encoded currents in the presence or absence of KN93, a potent pharmacological inhibitor of CaMKII. To this end, HEK-Nav1.2 cells were transiently transfected with *Gfp* or *Fgf12b-Gfp* and treated with either DMSO (0.05% final concentration) or KN93 (5 μm, final concentration) 30–60 min prior to the experiments. We found that GFP expressing HEK-Nav1.2 cells treated with KN93 exhibited significantly lower Na^+^ current (I_Na_) amplitudes than cells treated with DMSO (Fig 6*B–C*, black *versus* orange; Table I) or expressing FGF12B (Fig 6*B*–6*C*, black *versus* blue; Table I). This modulation confirms a pivotal role of CaMKII in regulating Nav currents, but adds new information on an isoform-specific mechanism of action of the kinase in that, compared with Nav1.5 cardiac sodium channels, CaMKII exerted effects also on Nav1.2-encoded current amplitude (53). No changes of I_Na_ amplitudes were found in the presence of FGF12B-GFP (Fig 6*B*–6*C*, *gray bar*) compared with GFP expressing cells treated with DMSO (Fig 6*B*–6*C*, *black bar*). We then analyzed the effect of FGF12B-GFP expression and KN93 on basic biophysical properties of Nav1.2 channels. Analysis of voltage-dependence of activation and steady-state inactivation revealed that treatment with KN93 in cells expressing GFP did not result in any significant shifts of either the V_½_ of activation (Fig. 6*D*–6*E*, gray *versus* blue; Table I) or V_½_ of steady-state inactivation compared with GFP expressing cells (Fig. 6*F–*6*G*, black *versus* blue; Table I). Likewise, Nav1.2-encoded currents in cells expressing FGF12B-GFP in DMSO did not differ from GFP control (Fig. 6*D*–6*G*, black *versus* gray*;* Table I). Notably, though, in cells expressing FGF12B-GFP and treated with KN93 the V_½_ of activation was significantly more depolarized compared with FGF12B-GFP in (Fig. 6*D*–6*E*, gray *versus* blue; Table I). Furthermore, cells expressing FGF12B-GFP and treated with KN93 exhibited a hyperpolarizing shift in the V_½_ of steady-state inactivation compared with GFP expressing cells (Fig. 6*F*–6*G*, black *versus* blue; Table I). Thus, FGF12B acts synergistically with the CaMKII signaling pathway to modulate Nav1.2 channel kinetics confirming that iFGF effects on Nav currents are phosphorylation-dependent.

**Table I:** Voltage-gated Na^+^ currents in HEK-Nav1.2 cells

Condition	Peak density (pA/pF)	Activation, V_1/2_ (mV)	*k*_act_ (mV)	Inactivation, V_1/2_ (mV)	*K*_inact_ (mV)
GFP (DMSO control)	−120.56 ± 20.05 (7)*^a^*	−16.84 ± 1.00 (7)	5.01 ± 0.64 (7)	−43.22 ± 1.74 (7)*^d^*	5.87 ± 0.32 (7)
GFP (KN93)	−49.34 ± 5.53 (8)*^a^*	−17.80 ± 1.14 (8)	4.34 ± 0.354 (8)	−46.64 ± 0.6 (8)	5.65 ± 0.43 (8)
FGF12-GFP (DMSO control)	−100.42 ± 14.65 (15)*^b^*	−18.88 ± 1.34 (14)*^c^*	4.55 ± 0.32 (14)	−46.70 ± 1.12 (12)	4.99 ± 0.19 (12)
FGF12-GFP (KN93)	−46.99 ± 9.46 (12)*^b^*	−13.88 ± 1.08 (12)*^c^*	4.95 ± 0.4 (12)	−50.47 ± 1.02 (11)*^d^*	4.77 ± 0.31 (11)

Analysis of voltage-dependences of I_Na_ amplitude, activation, and steady-state inactivation across conditions.

*^a,b^ p* < 0.05 by One-way ANOVA (Kruskal Wallis, *post hoc* analysis by Dunn's Method).

*^c,d^ p* < 0.05 by One-way ANOVA (*post hoc* analysis by Bonferroni).

## DISCUSSION

Emerging evidence indicates that stable and transient protein–protein interactions modulate the assembly, function, and stability of ion channel macromolecular complexes (5–9). However, the protein constituents of these complexes remain largely unknown except for a few recent studies (20–25). To this end, we set out to uncover Nav1.2 interacting proteins in an unbiased manner using affinity purification and quantitative proteomics. Ideally, we would guard against antibody off-target effects using Nav1.2 knockout animals (22, 25). However, no such model exists at present because complete knockout of the channel results in perinatal death (33). Thus, we chose to utilize isoform specific antiNav1.2 antibody (K69/3; UC Davis/NIH NeuroMab Facility, CA), which has been validated for target specificity in previous proteomic studies (28). We compared protein abundances between Nav1.2 affinity purifications from adult mammalian brain and an equal number of negative control purifications from the same biological samples. We evaluated proteins significantly enriched in Nav1.2 purifications compared with control purifications. We identified known interactors β1 (not quantified) and β2 subunits (3, 11), as well as Ankryin-3 (38, 39), calmodulin, and CaMKII (supplemental Table S4) throughout AP-MS methodology, which lends strength to the integrity of our data set. Though a previously reported interactor (*e.g.* synaptotagmin-1) (55) was not obtained in our results, which may have been because of the nature of interaction (*i.e.* more dynamic or lower affinity).

Otherwise, in this study we further examined the role of FGF12 a prominent interactor in our data set with ∼30-fold enrichment index and its modulation of Na^+^ currents. Previous studies in heterologous expression systems have linked FGF12 to cardiac Nav1.5 (56) and to Nav1.2 (57). Supporting this, confocal analysis showed for the first time to the best of our knowledge Nav1.2 and FGF12 complex formation in the AIS demonstrating colocalization of the two intercators in the native tissue at a subcellular location required for action potential initiation. Our co-IP experiments with myc-FGF12 were reciprocal to the AP-MS conducted *ex vivo* where FGF12 was precipitated with Nav1.2. Here Nav1.2 precipitated with myc-FGF12 validating both our mass spectrometry data and confocal analysis in native tissue. As a functional validation of our proteomic analysis, confocal imaging, and co-immunoprecipitation studies we applied patch-clamp electrophysiology to characterize the effect of FGF12B and the interactions on Nav1.2 currents using KN93 a potent CaMKII inhibitor. CaMKII is known to modulate Nav1.5 through phosphorylation (53). Notably, calmodulin, the primary substrate of CaMKII, has been crystalized in complex with FGF13 and the Nav1.5 C-tail (58) and both calmodulin and CaMKII were identified in our AP-MS studies, providing relevance of a 3-way interaction between iFGF and the calmodulin-CaMKII signaling complex in native conditions. The choice of including a CaMKII inhibitor in our study was also dictated by previous knowledge on iFGF modulation of Nav channels is phosphorylation-dependent (5, 44, 54).

We found that KN93 alone greatly suppressed Nav1.2 current amplitudes, but had no effects on the biophysical properties of Nav1.2, while we found no significant effects of FGF12B expression alone on Nav1.2 current amplitude of V_½_ of activation and inactivation. Notably, though, significant changes in the V_½_ of activation or steady-state inactivation were found in cells expressing FGF12B and treated with KN93. The V_½_ of activation was shifted to a more depolarized value in the FGF12B + KN93 category, whereas a hyperpolarizing shift was found for V_½_ of inactivation in the same cell group (FGF12B + KN93). Collectively, our results provide evidence for a modulation of Nav1.2 by CaMKII, which is a novel result in line with previous findings reported for Nav1.5, and by FGF12 via CaMKII. We show that CaMKII-dependent phosphorylation plays a critical role in regulating the amplitude independently of FGF12B and that the factor and the kinase act synergistically in controlling the channel kinetics. The lack of effect of FGF12B on Nav1.2 currents is in contradiction with the study by Wang *et al.*, (57), reporting FGF12B-dependent potentiation and hyperpolarizing shift of V_½_ of inactivation of Nav1.2 currents. This apparent discrepancy requires further investigation, but might be reconciled with different membrane trafficking routes and/or post-translational modifications of the Nav1.2 channel whether transfected transiently, as in Wang *et al.*, (57) or expressed stably as in our study. Altogether, these results emphasize that the effect of iFGF on Nav currents is cell background dependent and is dictated by the level of phosphorylation (5, 13, 54, 59, 60).

Inspection of the primary amino acid sequence of FGF12B and Nav1.2 reveals three and nine CaMKII phosphorylation motifs, respectively, but further studies are required to validate these potential Ser/Thr sites as CaMKII substrates. The KN93 reduction of Nav1.2 current amplitudes might be reconciled with reduced phosphorylation of S/T sites of the Nav1.2 channel that are functionally separated from the FGF12B binding site (proximal region of the channel C-tail). Candidate phosphorylation sites for this phenotype may lie within the N terminus or the I-II loop of Nav1.2 (supplemental Fig. S4). The synergistic action of FGF12B and KN93 on the channel kinetics might involve sites spatially close to the FGF12B binding site in the C-tail (28). However, further studies are needed to dissect the relative contribution of the various identified CaMKII isoforms (α, β, γ, and δ) to the functional modulation of Nav1.2 alone and Nav1.2:FGF12 complex. As at present, it remains unknown whether FGF12 or Nav1.2 are the primary target of CaMKII or whether any intermediate proteins/signaling molecules found in our data set are required.

In addition to CaMKII, we have previously shown that intracellular FGFs such as FGF14 are regulated in a phosphorylation-dependent manner through a GSK3 centered kinase network that includes Akt, PKC, and Wee1, kinases with known, important roles in maintaining neuronal polarity, modulating ion channel function, and regulating neuronal survival (5, 44). Kinase regulation of AIS proteins including iFGFs, spectrin (61), Nav channels (50–52), and voltage-gated potassium channels (62) have been shown to contribute to critical interactions with other AIS proteins, axonal development, and cytoskeletal integrity (15). Intracellular FGFs, in addition to their known role in regulation of Nav channels at the AIS (13, 60), also modulate members of the presynaptic Cav2 Ca^2+^ channel family (63) as well as Nav channels in the nodes of Ranvier (64, 65), suggesting that the role of iFGFs in modulating channel currents extends outside the AIS with broader implications for brain function.

Overall, the present findings provide an initial molecular framework for advancing our understanding of the Nav1.2 functional-structural interactions and promote further such investigations in the CNS.

## Supplementary Material

Supplemental Data

## Footnotes

^1^ The abbreviations used are:

Nav: voltage-gated sodium channel
CNS: central nervous system
FGF12: fibroblast growth factor 12
CAMKII: Ca2+/calmodulin-dependent protein kinase II
AIS: axonal initial segment
AP: affinity purification
CME: crude membrane extract
FDR: false discovery rate
