# Supplementary material for: Quantitative Proteomics Reveals Protein–Protein Interactions with Fibroblast Growth Factor 12 as a Component of the Voltage-Gated Sodium Channel 1.2 (Nav1.2) Macromolecular Complex in Mammalian Brain
Source: Mol Cell Proteomics. 2015 Feb 27;14(5):1288–300. doi: 10.1074/mcp.M114.040055 (PMC4424400; doi:10.1074/mcp.M114.040055)
Supplement: Supplemental Data [file supp_14_5_1288__index.html]

Quantitative Proteomics Reveals Protein-Protein Interactions with Fibroblast Growth Factor12 as a Component of the Nav1.2 Macromolecular Complex in Mammalian Brain — Quantitative Proteomics Reveals Protein–Protein Interactions with Fibroblast Growth Factor 12 as a Component of the Voltage-Gated Sodium Channel 1.2 (Nav1.2) Macromolecular Complex in Mammalian Brain — Mass Spectrometry Reveals Nav1.2 Protein–Protein Interaction — Supplemental Data 

# Quantitative Proteomics Reveals Protein–Protein Interactions with Fibroblast Growth Factor 12 as a Component of the Voltage-Gated Sodium Channel 1.2 (Nav1.2) Macromolecular Complex in Mammalian Brain

## Supplemental Data

**Files in this Data Supplement:**

- Supplemental Figure 1 - Supplemental Figure 1
- Supplemental Figure 2 - Supplemental Figure 2
- Supplemental Figure 3 - Supplemental Figure 3
- Supplemental Figure 4 - Supplemental Figure 4
- Supplemental Figure Legends - Supplemental Figure Legends
- Supplemental Table 4 - Supplemental Table 4
- Supplemental Table 1 - Supplemental Table 1
- Supplemental Table 2 - Supplemental Table 2
- Supplemental Table 3 - Supplemental Table 3
- Supplemental Table 5 - Supplemental Table 5
