# Supplementary material for: Quantitative Proteomics Reveals Protein–Protein Interactions with Fibroblast Growth Factor 12 as a Component of the Voltage-Gated Sodium Channel 1.2 (Nav1.2) Macromolecular Complex in Mammalian Brain
Source: Mol Cell Proteomics. 2015 Feb 27;14(5):1288–300. doi: 10.1074/mcp.M114.040055 (PMC4424400; doi:10.1074/mcp.M114.040055)
Supplement: Supplemental Data [file supp_M114.040055_mcp.M114.040055-2.pdf]

```
1     MARSVLVPPG PDSFRFFTRE SLAAIEQRIA EEKAKRPKQE RKDEDDENGP KPNSDLEAGK
                                 N-Terminus
61    SLPFIYGDIP PEMVSEPLED LDPYYINKKT FIVLNKGKAI SRFSATSALY ILTPFNPIRK
                                 N-Terminus
121   LAIKILVHSL FNVLIMCTIL TNCVFMTMSN PPDWTKNVEY TFTGIYTFES LIKILARGFC
                 ISI                                 IS2
181   LEDFTFLRNP WNWLDFTVIT FAYVTEFVNL GNVSALRTFR VLRALKTISV IPGLKTIVGA
                 IS3                                 IS4
241   LIQSVKKLSD VMILTVFCLS VFALIGLQLF MGNLRNKCLQ WPPDNSTFEI NITSFFNNSL
                             IS5
301   DWNGTAFNRT VNMFNWDEYI EDKSHFYFLE GQNDALLCGN SSDAGQCPEG YICVKAGRNP
361   NYGYTSFDTF SWAFLSLFRL MTQDFWENLY QLTLRAAGKT YMIFFVLVIF LGSFYLINLI
                                                       IS6
421   LAVVAMAYEE QNQATLEEAE QKEAEFQQML EQLKKQQEEA QAAAAAASAE SRDFSGAGGI
481   GVFSESSSVA SKLSSKSEKE LKNRRKKKKQ KEQAGEEEKE DAVRKSASED SIRKKGFQFS
541   LEGSRLTYEK RFSSPHQSLL SIRGSLFSPR RNSRASLFNF KGRVKDIGSE NDFADDEHST
601   FEDNDSRRDS LFVPHRHGER RPSNVSQASR ASRGIPTLPM NGKMHSAVDC NGVVSLVGGP
661   SALTSPVGQL LPEGTTTETE IRKRRSSSYH VSMDLLEDPS RQRAMSMASI LTNTMEELEE
721   SRQKCPPCWY KFANMCLIWD CCKPWLKVKH VVNLVVMDPF VDLAITICIV LNTLFMAMEH
                                                 IIS1
781   YPMTEQFSSV LSVGNLVFTG IFTAEMFLKI IAMDPYYYFQ EGWNIFDGFI VSLSLMELGL
                      IIS2                                  IIS3
841   ANVEGLSVLR SFRLLRVFKL AKSWPTLNML IKIIGNSVGA LGNLTLVLAI IVFIFAVVGM
                      IIS4                                  IIS5
901   QLFGKSYKEC VCKISNDCEL PRWHMHHFFH SFLIVFRVLC GEWIETMWDC MEVAGQTMCL
961   TVFMMVMVIG NLVVLNLFLA LLLSSFSSDN LAATDDDNEM NNLQIAVGRM QKGIDFVKRK
                 IIS6
1021  IREFIQKAFV RKQKALDEIK PLEDLNNKKD SCISNHTTIE IGKDLNYLKD GNGTTSGIGS
1081  SVEKYVVDES DYMSFINNPS LTVTVPIALG ESDFENLNTE EFSSESDMEE SKEKLNATSS
1141  SEGSTVDIGA PAEGEQPEAE PEESLEPEAC FTEDCVRKFK CCQISIEEGK GKLWWNLRKT
1201  CYKIVEHNWF ETFIVFMILL SSGALAFEDI YIEQRKTIKT MLEYADKVFT YIFILEMLLK
                 IIIS1                                      IIIS2
1261  WVAYGFQMYF TNAWCWLDFL IVDVSLVSLT ANALGYSELG AIKSLRTLRA LRPLRALSRF
                      IIIS3                            IIIS4
1321  EGMRVVVNAL LGAIPSIMNV LLVCLIFWLI FSIMGVNLFA GKFYHCINYT TGEMFDVSVV
                                      IIIS5
1381  NNYSECQALI ESNQTARWKN VKVNFDNVGL GYLSLLQVAT FKGWMDIMYA AVDSRNVELQ
1441  PKYEDNLYMY LYFVIFIIFG SFFTLNLFIG VIIDNFNQQK KKFGGQDIFM TEEQKKYYNA
                     IIIS6
1501  MKKLGSKKPQ KPIPRPANKF QGMVFDFVTK QVFDISIMIL ICLNMVTMMV ETDDQSQEMT
                                           IVS1
1561  NILYWINLVF IVLFTGECVL KLISLRHYYF TIGWNIFDFV VVILSIVGMF LAELIEKYFV
                 IVS2                            IVS3
1621  SPTLFRVIRL ARIGRILRLI KGAKGIRTLL FALMMSLPAL FNIGLLLFLV MFIYAIFGMS
                 IVS4                                       IVS5
1681  NFAYVKREVG IDDMFNFETF GNSMICLFQI TTSAGWDGLL APILNSGPPD CDPEKDHPGS
1741  SVKGDCGNPS VGIFFFVSYI IISFLVVVNM YIAVILENFS VATEESAEPL SEDDFEMFYE
                             IVS6
1801  VWEKFDPDAT QFIEFCKLSD FAAALDPPLL IAKPNKVQLI AMDLPMVSGD RIHCLDILFA
1861  FTKRVLGESG EMDALRIQME ERFMASNPSK VSYEPITTTL KRKQEEVSAI VIQRAYRRYL
1921  LKQKVKKVSS IYKKDKGKED EGTPIKEDII TDKLNENSTP EKTDVTPSTT SPPSYDSVTK
                                  C-Terminus
1981  PEKEKFEKDK SEKEDKGKDI RESKK
            C-Terminus
```
