# Supplementary material for: Quantitative Proteomics Reveals Protein–Protein Interactions with Fibroblast Growth Factor 12 as a Component of the Voltage-Gated Sodium Channel 1.2 (Nav1.2) Macromolecular Complex in Mammalian Brain
Source: Mol Cell Proteomics. 2015 Feb 27;14(5):1288–300. doi: 10.1074/mcp.M114.040055 (PMC4424400; doi:10.1074/mcp.M114.040055)
Supplement: Supplemental Data [file supp_M114.040055_mcp.M114.040055-3.pdf]

A

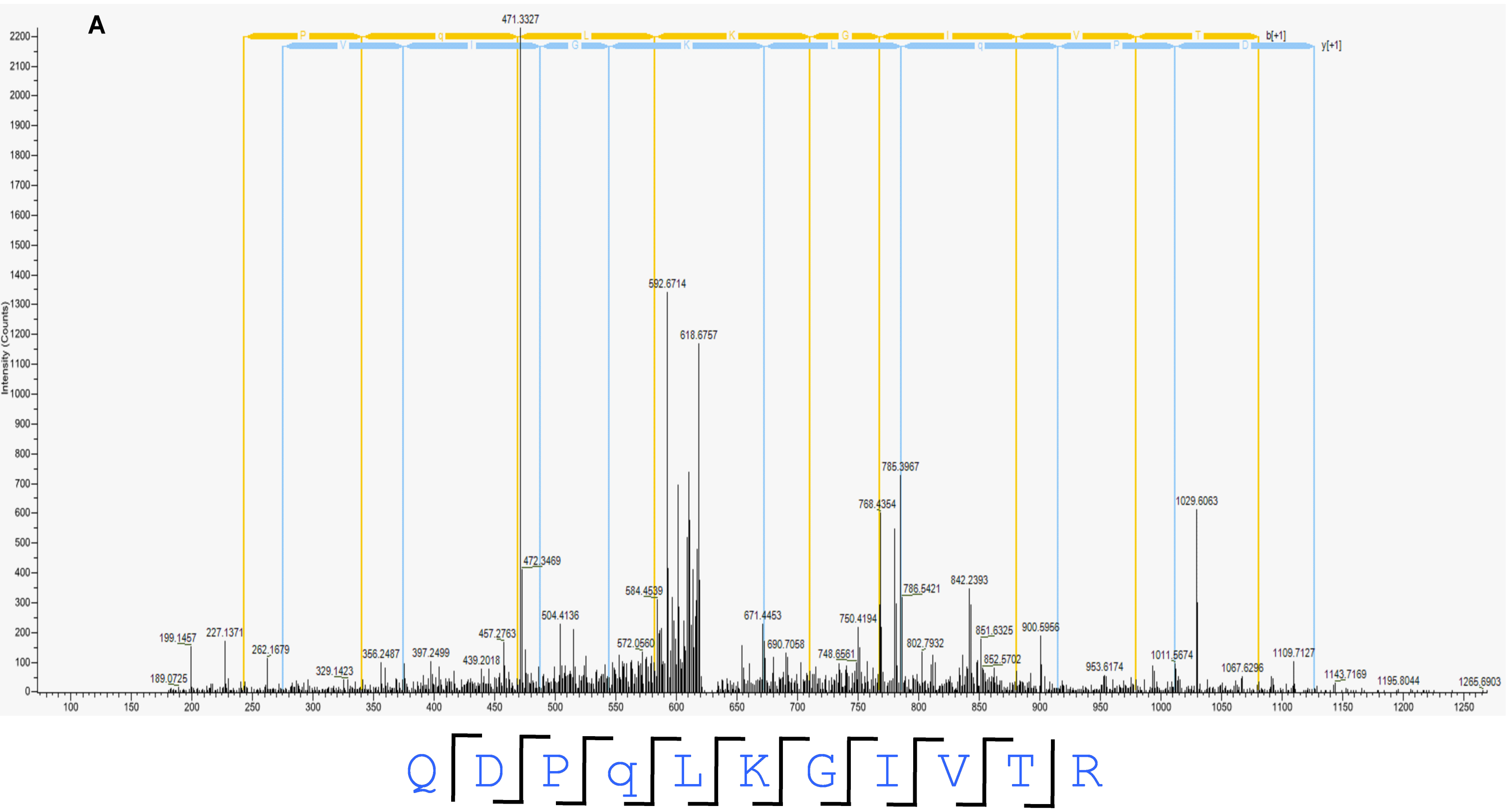

	(NH3+) -	D> <P	P> <q	q> <L	L> <K	K>	G> <I	I> <V	V>	T> <R	R - (COOH)
b (+1)	129.06586	244.09281	341.14558	470.18817	583.27224	711.36721	768.38868	881.47275	980.54117	1081.58885	1237.68997
b*(+1)	112.03931	227.06626	324.11903	453.16162	566.24569	694.34066	751.36213	864.44620	963.51462	1064.56230	1220.66342
b°(+1)	111.05529	226.08224	323.13501	452.17761	565.26168	693.35665	750.37812	863.46219	962.53061	1063.57829	1219.67941
y (+1)	1127.64196	1012.61501	915.56224	786.51964	673.43557	545.34060	488.31913	375.23506	276.16664	175.11896	1255.70054
y*(+1)	1110.61541	995.58846	898.53569	769.49309	656.40902	528.31405	471.29258	358.20851	259.14009	158.09241	1238.67399
y°(+1)	1109.63139	994.60444	897.55167	768.50908	655.42501	527.33004	470.30857	357.22450	258.15608	157.10840	1237.68997

B

```
1     MAAAIASSLIRQKRQARESNSDRVSASKRRSSPSKDGRSLCERHVLGVFSKVRFCSGRKR    60   P61329    FGF12_MOUSE
1     MAAAIASGLIRQKRQAREQHWDRPSASRRRSSPSKN-RGLFNGNLVDIFSKVRIFGLKKR    59   P70379    FGF14_MOUSE
1     MAAAIASGLIRQKRQAREQHWDRPSASRRRSSPSKN-RGLCNGNLVDIFSKVRIFGLKKR    59   Q8R5L7    FGF14_RAT
1     MAAAIASSLIRQKRQARESNSDRVSASKRRSSPSKDGRSLCERHVLGVFSKVRFCSGRKR    60   P61150    FGF12_RAT

61    PVRRRPEPQLKGIVTRLFSQQGYFLQMHPDGTIDGTKDENSDYTLFNLIPVGLRVVAIQG   120   P61329    FGF12_MOUSE
60    R-LRRQDPQLKGIVTRLYCRQGYYLQMHPDGALDGTKDDSTNSTLFNLIPVGLRVVAIQG   118   P70379    FGF14_MOUSE
60    R-LRRQDPQLKGIVTRLYCRQGYYLQMHPDGALDGTKDDSTNSTLFNLIPVGLRVVAIQG   118   Q8R5L7    FGF14_RAT
61    PVRRRPEPQLKGIVTRLFSQQGYFLQMHPDGTIDGTKDENSDYTLFNLIPVGLRVVAIQG   120   P61150    FGF12_RAT

121   VKASLYVAMNGEGYLYSSDVFTPECKFKESVFENYYVIYSSTLYRQQESGRAWFLGLNKE   180   P61329    FGF12_MOUSE
119   VKTGLYIAMNGEGYLYPSELFTPECKFKESVFENYYVIYSSMLYRQQESGRAWFLGLNKE   178   P70379    FGF14_MOUSE
119   VKTGLYIAMNGEGYLYPSELFTPECKFKESVFENYYVIYSSMLYRQQESGRAWFLGLNKE   178   Q8R5L7    FGF14_RAT
121   VKASLYVAMNGEGYLYSSDVFTPECKFKESVFENYYVIYSSTLYRQQESGRAWFLGLNKE   180   P61150    FGF12_RAT

181   GQIMKGNRVKKTKPSSHFVPKPIEVCMYREPSLHEIGEKQGRS-----RKSSGTPTMNGG   235   P61329    FGF12_MOUSE
179   GQVMKGNRVKKTKPAAHFLPKPLEVAMYREPSLHDVGETVPKAGVTPSKSTSASAIMNGG   238   P70379    FGF14_MOUSE
179   GQVMKGNRVKKTKPAAHFLPKPLEVAMYREPSLHDVGETVPKAGVTPSKSTSASAIMNGG   238   Q8R5L7    FGF14_RAT
181   GQIMKGNRVKKTKPSSHFVPKPIEVCMYREPSLHEIGEKQGRS-----RKSSGTPTMNGG   235   P61150    FGF12_RAT

236   KVVNQDST-   243   P61329    FGF12_MOUSE
239   KPVNKCKTT   247   P70379    FGF14_MOUSE
239   KPVNKCKTT   247   Q8R5L7    FGF14_RAT
236   KVVNQDST-   243   P61150    FGF12_RAT
```
