# Supplementary material for: Quantitative Proteomics Reveals Protein–Protein Interactions with Fibroblast Growth Factor 12 as a Component of the Voltage-Gated Sodium Channel 1.2 (Nav1.2) Macromolecular Complex in Mammalian Brain
Source: Mol Cell Proteomics. 2015 Feb 27;14(5):1288–300. doi: 10.1074/mcp.M114.040055 (PMC4424400; doi:10.1074/mcp.M114.040055)
Supplement: Supplemental Data [file supp_M114.040055_mcp.M114.040055-4.pdf]

>sp|P61150-2|FGF12_RAT Isoform 2 of Fibroblast growth factor 12 OS=Rattus norvegicus GN=Fgf12

```
MESKEPQLKG IVTRLFSQQG YFLQMHPDGT IDGTKDENSD YTLFNLIPVG    50
LRVVAIQGVK ASLYVAMNGE GYLYSSDVFT PECKFKESVF ENYYVIYSST   100
LYRQQESGRA WFLGLNKEGQ IMKGNRVKKT KPSSHFVPKP IEVCMYREPS   150
LHEIGEKQGR SRKSSGTPTM NGGKVVNQDS T                       181
```

>sp|P61328-2|FGF12_HUMAN Isoform 2 of Fibroblast growth factor 12 OS=Homo sapiens GN=FGF12

```
MESKEPQLKG IVTRLFSQQG YFLQMHPDGT IDGTKDENSD YTLFNLIPVG    50
LRVVAIQGVK ASLYVAMNGE GYLYSSDVFT PECKFKESVF ENYYVIYSST   100
LYRQQESGRA WFLGLNKEGQ IMKGNRVKKT KPSSHFVPKP IEVCMYREPS   150
LHEIGEKQGR SRKSSGTPTM NGGKVVNQDS T                       181
```

>sp|P04775|SCN2A_RAT Sodium channel protein type 2 subunit alpha OS=Rattus norvegicus GN=Scn2a PE=1 SV=1

```
1     MARSVLVPPG PDSFRFFTRE SLAAIEQRIA EEKAKRPKQE RKDEDDENGP KPNSDLEAGK
                                   N-Terminus
61    SLPFIYGDIP PEMVSEPLED LDPYYINKKT FIVLNKGKAI SRFSATSALY ILTPFNPIRK
                                   N-Terminus
121   LAIKILVHSL FNVLIMCTIL TNCVFMTMSN PPDWTKNVEY TFTGIYTFES LIKILARGFC
                    ISI                                 IS2
181   LEDFTFLRNP WNWLDFTVIT FAYVTEFVNL GNVSALRTFR VLRALKTISV IPGLKTIVGA
                    IS3                                 IS4
241   LIQSVKKLSD VMILTVFCLS VFALIGLQLF MGNLRNKCLQ WPPDNSTFEI NITSFFNNSL
                          IS5
301   DWNGTAFNRT VNMFNWDEYI EDKSHFYFLE GQNDALLCGN SSDAGQCPEG YICVKAGRNP
361   NYGYTSFDTF SWAFLSLFRL MTQDFWENLY QLTLRAAGKT YMIFFVLVIF LGSFYLINLI
                                                              IS6
```

421 LAVVAMAYEE QNQATLEEAE QKEAEFQQML EQLKKQQEEA QAAAAAASAE S**RDFS**GAGGI

481 GVFSESSSVA SKLSSKSEKE LKNRRKKKKQ KEQAGEEEKE DAVRKSASED SIRKKGFQFS

541 LEGSRLTYEK **RFSS**PHQSLL SIRGSLFSP**R RNS**RASLFNF KGRVKDIGSE NDFADDEHST

601 FEDNDS**RRDS** LFVPHRHGE**R** **RPS**NVSQASR ASRGIPTLPM NGKMHSAVDC NGVVSLVGGP

661 SALTSPVGQL LPEGTTTETE IRKR**RSSS**YH VSMDLLEDPS RQ**RAMS**MASI LTNTMEELEE

721 SRQKCPPCWY KFANMCLIWD CCKPWLKVKH VVNLVVMDPF VDLAITICIV LNTLFMAMEH

IIS1

781 YPMTEQFSSV LSVGNLVFTG IFTAEMFLKI IAMDPYYYFQ EGWNIFDGFI VSLSLMELGL

IIS2 **IIS3**

841 ANVEGLSVLR SFRLLRVFKL AKSWPTLNML IKIIGNSVGA LGNLTLVLAI IVFIFAVVGM

IIS4 **IIS5**

901 QLFGKSYKEC VCKISNDCEL PRWHMHHFFH SFLIVFRVLC GEWIETMWDC MEVAGQTMCL

961 TVFMMVMVIG NLVVLNLFLA LLLSSFSSDN LAATDDDNEM NNLQIAVGRM QKGIDFVKRK

IIS6

1021 IREFIQKAFV RKQKALDEIK PLEDLNNKKD SCISNHTTIE IGKDLNYLKD GNGTTSGIGS

1081 SVEKYVVDES DYMSFINNPS LTVTVPIALG ESDFENLNTE EFSSESDMEE SKEKLNATSS

1141 SEGSTVDIGA PAEGEQPEAE PEESLEPEAC FTEDCVRKFK CCQISIEEGK GKLWWNLRKT

1201 CYKIVEHNWF ETFIVFMILL SSGALAFEDI YIEQRKTIKT MLEYADKVFT YIFILEMLLK

IIIS1 **IIIS2**

1261 WVAYGFQMYF TNAWCWLDFL IVDVSLVSLT ANALGYSELG AIKSLRTLRA LRPL**RALS**RF

IIIS3 **IIIS4**

1321 EGMRVVVNAL LGAIPSIMNV LLVCLIFWLI FSIMGVNLFA GKFYHCINYT TGEMFDVSVV

IIIS5

1381 NNYSECQALI ESNQTARWKN VKVNFDNVGL GYLSLLQVAT FKGWMDIMYA AVDSRNVELQ

1441 PKYEDNLYMY LYFVIFIIFG SFFTLNLFIG VIIDNFNQQK KKFGGQDIFM TEEQKKYYNA

IIIS6

1501 MKKLGSKKPQ KPIPRPANKF QGMVFDFVTK QVFDISIMIL ICLNMVTMMV ETDDQSQEMT

IVS1

1561 NILYWINLVF IVLFTGECVL KLISLRHYYF TIGWNIFDFV VVILSIVGMF LAELIEKYFV

IVS2 **IVS3**

1621 SPTLFRVIRL ARIGRILRLI KGAKGIRTLL FALMMSLPAL FNIGLLLFLV MFIYAIFGMS

IVS4 **IVS5**

1681 NFAYVKREVG IDDMFNFETF GNSMICLFQI TTSAGWDGLL APILNSGPPD CDPEKDHPGS

1741 SVKGDCGNPS VGIFFFVSYI IISFLVVVNM YIAVILENFS VATEESAEPL SEDDFEMFYE

IVS6

1801 VWEKFDPDAT QFIEFCKLSD FAAALDPPLL IAKPNKVQLI AMDLPMVSGD RIHCLDILFA

1861 FTKRVLGESG EMDALRIQME ERFMASNPSK VSYEPITTTL KRKQEEVSAI VIQRAYRRYL

1921 LKQKVKKVSS IYKKDKGKED EGTPIKEDII TDKLNENSTP EKTDVTPSTT SPPSYDSVTK

C-Terminus

1981 PEKEKFEKDK SEKEDKGKDI RESKK

C-Terminus
