# Supplementary material for: Quantitative Proteomics Reveals Protein–Protein Interactions with Fibroblast Growth Factor 12 as a Component of the Voltage-Gated Sodium Channel 1.2 (Nav1.2) Macromolecular Complex in Mammalian Brain
Source: Mol Cell Proteomics. 2015 Feb 27;14(5):1288–300. doi: 10.1074/mcp.M114.040055 (PMC4424400; doi:10.1074/mcp.M114.040055)
Supplement: Supplemental Data [file supp_M114.040055_mcp.M114.040055-5.pdf]

Supplemental Figures

Figure S1. Mass measurement error distribution and principle component analysis of nLC-MS/MS data. (*A*) Frequency of precursor mass error in ppm of all peptides identified by our combined database search annotated at ≥ 95% probability. All peptides fall within ±5 ppm. (*B*) Principal component analysis of all biological and technical replicates across experimental conditions. Nav1.2 pulldowns are shown in red while controls are shown in blue. The letters X, Y, and Z designate distinct biological replicates. Numbers correspond to the technical replicate (i.e. individual nL-MS/MS run).

Figure S2. Amino acid sequence coverage obtained for the canonical form of Nav1.2 (Uniprot accession number P04775). Identified peptides are highlighted in yellow. Underlined sequences represent predicted transmembrane α-helices (S1-S6) in each domain (I-IV).

Figure S3. Mass spectral analysis of FGF14. (*A*) Annotated MS/MS spectra of the only unique FGF14 peptide found in our dataset with corresponding ion table below. This peptide is also deamidated at the second glutamine (q) leading us to remove this protein from quantification. (*B*) Sequence alignment between FGF12 rat and mouse and FGF14 rat and mouse show that the peptide QDPqLKGIVTR is unique to FGF14 in both species.

Figure S4. Amino acid sequence of FGF12B rat and human (Uniprot accession number P61150-2 and P61328-2) showing the minimum CaMKII phosphorylation motifs in yellow with Nav1.2 (Uniprot accession number P04775) shown below.
