# Supplementary material for: Quantitative Proteomics Reveals Protein–Protein Interactions with Fibroblast Growth Factor 12 as a Component of the Voltage-Gated Sodium Channel 1.2 (Nav1.2) Macromolecular Complex in Mammalian Brain
Source: Mol Cell Proteomics. 2015 Feb 27;14(5):1288–300. doi: 10.1074/mcp.M114.040055 (PMC4424400; doi:10.1074/mcp.M114.040055)
Supplement: Supplemental Data [file supp_M114.040055_mcp.M114.040055-9.pdf]

Supplementary table 4. Protein interactors of the Nav1.2 channel in rat brain

Accession	Protein ID	*p*-value	*q*-value	Log_2 (Nav/Con)
O54774	AP-3 complex subunit delta-1	<.0001	0.0001	4.4812
P11442	Clathrin heavy chain 1	<.0001	0.0003	3.4461
P04775	Nav1.2	<.0001	0.0004	4.6394
Q9WTP0	Band 4.1-like protein 1	<.0001	0.0008	4.6133
Q9JME5	AP-3 complex subunit beta-2	<.0001	0.0009	4.5329
P19945	60S acidic ribosomal protein P0	<.0001	0.0011	2.5640
P54900	Sodium channel subunit beta-2	<.0001	0.0012	5.7346
Q5FVM7	DnaJ homolog subfamily C member 16	<.0001	0.0015	4.5314
Q9DBG3	AP-2 complex subunit beta	<.0001	0.0016	1.2339
P53678	AP-3 complex subunit mu-2	<.0001	0.0018	3.2867
P62161	Calmodulin	<.0001	0.0019	4.4258
P21533	60S ribosomal protein L6	<.0001	0.0023	2.7159
P62718	60S ribosomal protein L18a	<.0001	0.0024	2.5151
P17426	AP-2 complex subunit alpha-1	<.0001	0.0026	1.6509
P12970	60S ribosomal protein L7a	<.0001	0.0030	3.3284
Q8BSZ2	AP-3 complex subunit sigma-2	<.0001	0.0032	4.6883
P17427	AP-2 complex subunit alpha-2	<.0001	0.0034	1.6689
Q6IRU5	Clathrin light chain B	<.0001	0.0036	3.1237
P06907	Myelin protein P0	<.0001	0.0042	4.0849
Q9D051	PDHE1-B	<.0001	0.0043	1.1468
P35979	60S ribosomal protein L12	<.0001	0.0046	1.6591
P62907	60S ribosomal protein L10a	<.0001	0.0047	2.5068
O08585	Clathrin light chain A	<.0001	0.0049	3.5721
P97379	G3BP-2	<.0001	0.0050	2.8594
P41123	60S ribosomal protein L13	<.0001	0.0051	2.0042
D4A631	BIG1	<.0001	0.0058	5.1843
P17702	60S ribosomal protein L28	<.0001	0.0059	1.5710
P02401	60S acidic ribosomal protein P2	<.0001	0.0065	2.7808
P13383	Nucleolin	<.0001	0.0066	1.9710
Q9DCR2	AP-3 complex subunit sigma-1	<.0001	0.0068	3.4381
P53676	AP-3 complex subunit mu-1	<.0001	0.0070	2.9341
P29341	Polyadenylate-binding protein 1	<.0001	0.0073	2.3641
Q9DBR3	Armadillo repeat-containing protein 8	<.0001	0.0082	6.5151
P29314	40S ribosomal protein S9	<.0001	0.0084	1.5098
Q05186	Reticulocalbin-1	<.0001	0.0086	2.5712
Q5U3K5	Rab-like protein 6	<.0001	0.0088	3.0331
P62902	60S ribosomal protein L31	<.0001	0.0093	1.1241
P62278	40S ribosomal protein S13	<.0001	0.0095	0.7890
P11798	CaMKII alpha	<.0001	0.0097	1.0137
P61329	Fibroblast growth factor 12	<.0001	0.0099	5.1215
Q4V8F9	HSDL2	<.0001	0.0104	3.7310
P86252	Transcriptional activator protein Pur-alpha	<.0001	0.0108	1.8087
Q9D7M1	GID8	<.0001	0.0112	4.2844
P02091	Hemoglobin subunit beta-1	<.0001	0.0114	1.0654
P43277	Histone H1.3	<.0001	0.0115	0.9817
Q63507	60S ribosomal protein L14	<.0001	0.0118	2.2051
P39032	60S ribosomal protein L36	<.0001	0.0119	1.8047
P06761	78 kDa glucose-regulated protein	<.0001	0.0120	0.5974
O55142	60S ribosomal protein L35a	<.0001	0.0122	2.4280
P0C1X8	AP2-associated protein kinase 1	<.0001	0.0127	1.3241
P35427	60S ribosomal protein L13a	<.0001	0.0128	2.8251
P97351	40S ribosomal protein S3a	<.0001	0.0132	0.9269
P61265	Syntaxin-1B	<.0001	0.0135	2.0956
P11881	Inositol 1,4,5-trisphosphate receptor type 1	<.0001	0.0138	1.3738
P61358	60S ribosomal protein L27	<.0001	0.0139	2.3643
P61979	Heterogeneous nuclear ribonucleoprotein K	<.0001	0.0142	0.9830
Q64478	Histone H2B type 1-H	<.0001	0.0145	1.1762
P62890	60S ribosomal protein L30	<.0001	0.0158	2.7368
P62918	60S ribosomal protein L8	<.0001	0.0159	2.0509
P16086	Spectrin alpha chain, non-erythrocytic 1	<.0001	0.0164	1.5687
P69897	Tubulin beta-5 chain	<.0001	0.0165	0.8034
Q00728	Histone H2A type 4	<.0001	0.0166	1.4264
P08413	CaMKII beta	<.0001	0.0170	1.4391
Q9Z1T1	AP-3 complex subunit beta-1	<.0001	0.0172	4.5027
P84586	hnRNP G	<.0001	0.0176	1.9331
P63045	Vesicle-associated membrane protein 2	<.0001	0.0177	2.0036
P34926	Microtubule-associated protein 1A	<.0001	0.0180	0.9688
D3ZBN0	Histone H1.5	<.0001	0.0193	0.8922
Q9D8B3	Charged multivesicular body protein 4b	<.0001	0.0196	2.1346
Q05175	Brain acid soluble protein 1	<.0001	0.0203	0.7407
P11517	Hemoglobin subunit beta-2	<.0001	0.0204	1.4579
P62806	Histone H4	<.0001	0.0207	0.8718
P60879	Synaptosomal-associated protein 25	<.0001	0.0208	1.5790
Q68A21	Transcriptional activator protein Pur-beta	<.0001	0.0212	1.9368
G5E8K5	Ankyrin-3	<.0001	0.0214	3.0766
P15146	Microtubule-associated protein 2	<.0001	0.0215	0.7653
P05426	60S ribosomal protein L7	<.0001	0.0216	1.1693
P27952	40S ribosomal protein S2	<.0001	0.0230	1.1756
Q3TEA8	Heterochromatin protein 1-binding protein 3	<.0001	0.0231	3.1852
P60203	Myelin proteolipid protein	<.0001	0.0235	0.7135
P35565	Calnexin	<.0001	0.0238	2.0720
P61982	14-3-3 protein gamma	<.0001	0.0242	0.9802
P68510	14-3-3 protein eta	<.0001	0.0246	1.1790
P11730	CaMKII gamma	<.0001	0.0250	0.6703
O09167	60S ribosomal protein L21	<.0001	0.0251	1.8768
P84089	Enhancer of rudimentary homolog	<.0001	0.0265	1.7073
P27274	CD59 glycoprotein	0.0001	0.0268	1.4231
Q6PDM2	Serine/arginine-rich splicing factor 1	0.0002	0.0272	1.3555
Q6ZQ08	CCR4-NOT transcription complex subunit 1	0.0002	0.0273	3.1433
P10111	Peptidyl-prolyl cis-trans isomerase A	0.0003	0.0277	1.0682
P62911	60S ribosomal protein L32	0.0003	0.0280	1.1588
P62855	40S ribosomal protein S26	0.0004	0.0284	0.8119
Q9WV55	VAMP-A	0.0004	0.0288	0.8569
P12960	Contactin-1	0.0006	0.0295	1.8618
P15205	Microtubule-associated protein 1B	0.0006	0.0296	0.6361
P63102	14-3-3 protein zeta/delta	0.0007	0.0297	0.7103
Q8K0T0	Reticulon-1	0.0011	0.0300	0.6230
P29457	Serpin H1	0.0042	0.0320	0.7308
P11598	Protein disulfide-isomerase A3	0.0068	0.0324	2.4193
A2A5R2	BIG2	0.0085	0.0328	2.5120
P16125	L-lactate dehydrogenase B chain	0.0092	0.0331	2.6435
P55063	Heat shock 70 kDa protein 1-like	0.0093	0.0332	0.5915
Q63425	Periaxin	0.0096	0.0334	2.7240
P35980	60S ribosomal protein L18	0.0165	0.0346	0.9719
P05065	Fructose-bisphosphate aldolase A	0.0190	0.0350	0.6144
O55100	Synaptogyrin-1	0.0290	0.0355	0.6514
Q66HD0	Endoplasmin	0.0484	0.0368	1.1760

Proteins identified in Nav1.2 purifications (**Fig. 1A** and **Fig. 2**) listed by *q*-value and logarithmized ratios of Nav *vs* control; accession refers to the UniProtKB database identifiers and *q*-value refers to the *p*-value corrected for multiple hypothesis testing using the Benjamini–Hochberg method where $q \leq 0.5$ is considered significant.
